# The effect of preventive use of corticosteroids on postoperative complications after esophagectomy: A retrospective cohort study

**DOI:** 10.1038/s41598-019-48349-0

**Published:** 2019-08-19

**Authors:** Heejoon Jeong, Ji Won Choi, Hyun Joo Ahn, Yong Soo Choi, Jie Ae Kim, Mikyung Yang, Jin Kyoung Kim, Duk Kyung Kim, Byung Seop Shin, Sang Hyun Lee, Young Ri Kim, Mihye Park, Yoon Joo Chung

**Affiliations:** 1 Department of Anesthesiology and Pain Medicine, Samsung Medical Center, Sungkyunkwan University School of Medicine, 81 Irwon-ro, Gangnam-gu, Seoul, 06351 Republic of Korea; 2 Department of Thoracic Surgery, Samsung Medical Center, Sungkyunkwan University School of Medicine, 81 Irwon-ro, Gangnam-gu, Seoul, 06351 Republic of Korea

**Keywords:** Risk factors, Oesophageal cancer

## Abstract

Corticosteroids have been empirically administered to reduce the rate of acute respiratory distress syndrome (ARDS) after esophagectomy. However, their efficacy remains controversial, and corticosteroids may increase the risk of graft dehiscence and infection, which are major concerns after esophagectomy. Therefore, we compared the incidence of composite complications (ARDS, graft dehiscence and infection) after esophagectomy between patients who received a preventive administration of corticosteroids and those who did not. All patients who underwent esophagectomy from 2010 to 2015 at a tertiary care university hospital were reviewed retrospectively (*n* = 980). Patients were divided into Steroid (*n* = 120) and Control (*n* = 860) groups based on the preventive administration of 100 mg hydrocortisone during surgery. The primary endpoint was the incidence of composite complications. The incidence of composite complications was not different between the Control and Steroid groups (17.4% vs. 21.7% respectively; *P* = 0.26). The incidence rates of complications in each category were not different between the Control and Steroid groups: ARDS (3.8% vs. 5.0%; *P* = 0.46), graft dehiscence (4.8% vs. 6.7%; *P* = 0.37), and infection (12.8% vs. 15.8%; *P* = 0.36). Propensity score matching revealed that composite complications (20.0% vs. 21.7%; *P* = 0.75), ARDS (4.3% vs. 5.2%; *P* = 0.76) and infection (16.5% vs. 15.7%; *P* = 0.86) were not different between the Control and Steroid group, but the incidence of graft dehiscence was higher in the Steroid group than in the Control group (0.9% vs. 7.0%; *P* = 0.0175). In conclusions, the preventive use of corticosteroids did not reduce the incidence of ARDS, but may be related to an increased incidence of graft dehiscence. Therefore, routine administration of corticosteroids to prevent ARDS is not recommended in esophagectomy.

## Introduction

Esophageal cancer is the eighth most common cancer and the sixth most common cause of cancer-related death^1^. Esophagectomy is the main therapeutic modality used to cure esophageal cancer but is a high-risk procedure^1^. The perioperative mortality rate was 3.4% and major morbidity occurred in 33.1% according to the Society of Thoracic Surgeons General Thoracic Surgery 2016 Database^2^.

Acute respiratory distress syndrome (ARDS) is a major cause of mortality and morbidity after esophagectomy^3,4^. The underlying mechanism of ARDS is the massive release of inflammatory cytokines. The radical dissection of gastro-enteral organs^5–7^, lung injury during the operation, and one-lung ventilation increase inflammatory cytokines in both the operated and non-operated lungs^8^. Excessive neutrophils recruited in response to the pro-inflammatory cytokines increase pulmonary vascular permeability^9^. These reactions often precede the systemic inflammatory response syndrome or ARDS^6,10^.

Corticosteroids inhibit the transcription of mRNA that encodes inflammatory cytokines, thus reducing acute-phase reactants and inflammation^11^. Therefore, corticosteroids have been used to suppress inflammatory reactions in many clinical conditions^12,13^, and empirically administered during esophagectomy to prevent ARDS^14,15^. However, some studies have reported no beneficial effect of administering corticosteroids during an esophagectomy^5,16^. Moreover, corticosteroids can impede the healing process of surgical wounds, resulting in leakage of the anastomosis site due to their anti-inflammatory effects and antagonistic effects on growth factors^12,17,18^. Abnormal immune defenses arising from the perioperative use of corticosteroids can also cause surgical site infection and may increase the incidence of pneumonia^12,19,20^. Graft dehiscence and infection (including pneumonia) are other major morbidities in patients undergoing esophagectomy^2–4,21–23^.

Due to the complex effects of corticosteroids, risk-benefit studies on their preventive use are required. However, few studies have assessed the composite complications (ARDS, graft dehiscence, infection) in patients undergoing esophagectomy. In this retrospective study, we reviewed our clinical data from a large group of patients who underwent esophagectomy to compare the incidence of composite complications until discharge between patients who received preventive administration of corticosteroids and those who did not.

## Results

**Figure 1:**
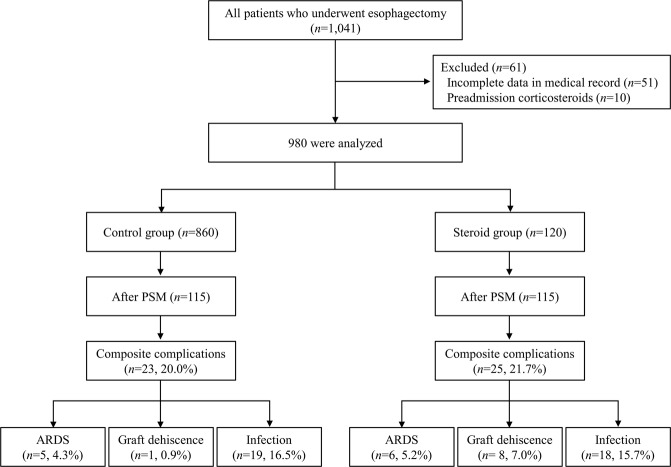
Flow diagram and the incidence of complications in each group. Abbreviation: PSM propensity score matched, ARDS acute respiratory distress syndrome.

A total of 1,041 patients received an esophagectomy in our institute between 2010 and 2015. Patients with incomplete data in their medical records (*n* = 51) and patients already receiving corticosteroids (*n* = 10) were excluded. Thus, the final analysis included 980 patients. Overall, 120 patients received corticosteroid (100 mg hydrocortisone) for preventive use and the remaining 860 patients were classified into the Control group (Fig. 1).

The demographic and clinical characteristics of the groups are shown in Table 1. No differences in demographics or underlying comorbidities were observed between the Control and Steroid groups, except in cardiac disease (5% vs.1%, Control vs. Steroid; *P* = 0.040). The Steroid group received more fluid (net fluid balance during operation and postoperative 24 h, median [interquartile range]: 381 mL [34 to 770] vs. 533 [158 to 986]; *P* = 0.006), transfusions (5% vs. 10%; *P* = 0.031), and thoracic epidural analgesia (35% vs. 45%; *P* = 0.033) than the Control group.

**Table 1:** Baseline characteristics of patients and operations.

Variables	Control (*n* = 860)	Steroid (*n* = 120)	*P* value
**Preoperative data**			
Age (years)	63 [57–70]	65 [60–70]	0.06
Male	786 (91)	109 (91)	0.84
Body mass index (kg/m^2^)	23 [20–25]	23 [21–25]	0.25
Hemoglobin (g/dl)	14 [12–15]	14 [13–15]	0.64
Albumin (g/dl)	4 [4–5]	4 [4–5]	0.52
ASA physical status ≥3	47 (6)	5 (4)	0.52
TNM Stage (*n* = 868)			
1	296 (39)	39 (37)	0.68
2	226 (30)	28 (26)	
3	230 (30)	38 (36)	
4	10 (1)	1 (1)	
Histologic grade (*n* = 800)			
Well differentiated	80 (11.5)	13 (12.6)	0.43
Moderately differentiated	501 (71.9.)	78 (75.7)	
Poorly differentiated	116 (16.6)	12 (1.5)	
Tumor location (*n* = 865)			
Upper third	45 (5.9)	6 (5.8)	0.65
Middle third	126 (16.6)	21 (20.2)	
Lower third	590 (77.5)	77 (74.0)	
Tumor length (*n* = 811)			
≤3 cm	387 (54.2)	57 (58.8)	0.16
3–6 cm	247 (34.6)	25 (25.8)	
>6 cm	80 (11.2)	15 (15.5)	
Neoadjuvant CCRT (*n* = 823)	91 (13.6)	21 (13.5)	0.95
Comorbid condition			
Hypertension	332 (39)	40 (33)	0.27
Diabetes mellitus	130 (15)	17 (14)	0.79
Pulmonary dysfunction^a^	253 (29)	39 (32)	0.49
Cardiac disease	43 (5)	1 (1)	0.0389
Cerebrovascular disease	42 (5)	7 (6)	0.66
Renal dysfunction	7 (1)	1 (1)	1.00
Intraoperative data			
Duration of surgery (min)	276 [237–348]	268 [237–355]	0.80
Type of surgery			
Ivor Lewis operation	480 (56)	74 (62)	0.16
Three fields operation	161 (19)	27 (23)	
Three holes operation	107 (12)	8 (7)	
Etc.^b^	51 (6)	5 (4)	
Net balance of fluid (ml)	381 [34–770]	533 [158–986]	0.0064
Infusion of inotrope	203 (24)	29 (24)	0.89
Infusion of vasopressor	485 (56)	79 (66)	0.05
Transfusion	44 (5)	12 (10)	0.0308
Thoracic epidural analgesia	301 (35)	54 (45)	0.0328
Hospital days (day)	13 [11–16]	12.5 [11–16]	0.75
ICU stay (day)	2 [2–3]	2 [2–3]	0.92

Values are presented as median [interquartile] or *n* (%). ^a^Pulmonary dysfunction included lung diseases (chronic obstructive pulmonary disease, bronchiectasis, asthma, interstitial lung disease), preoperative forced expiratory volume in one-second (FEV_1_) <60% of predicted value and current smoker; current smoker was defined as patients who kept smoking or stop smoking within 1 month before surgery. ^b^Etc. included esophagocolonogastrostomy, esophagocolonojejunostomy, transhiatal esophagectgomy, and total gastrectomy. Abbreviations: ASA American Society of Anesthesiologist, TNM tumor node metastasis, CCRT concurrent chemoradiotherapy, ICU intensive care unit.

The incidence of composite complications was 18% (ARDS 4%, graft dehiscence 5%, and infection including pneumonia 13%) in the overall population. The incidence of composite complications was not different between the groups (17.4% in the Control group and 21.7% in the Steroid group, *P* = 0.26) (Table 2). The incidence of complications in each category was not different between the Control and Steroid groups: ARDS (3.8% vs. 5.0%; *P* = 0.46), graft dehiscence (4.8% vs. 6.7%; *P* = 0.37), and infection (12.8% vs. 15.8%; *P* = 0.36) (Table 2).

**Table 2:** Postoperative complications between Control and Steroid groups.

Complication [*n* (%)]	Control (*n* = 860)	Steroid (*n* = 120)	*P* value
Composite complication	150 (17.4)	26 (21.7)	0.26
ARDS	33 (3.8)	6 (5.0)	0.46
Graft dehiscence	41 (4.8)	8 (6.7)	0.37
Infection	110 (12.8)	19 (15.8)	0.36
Other complications			
Chylothorax	27 (3.1)	4 (3.3)	0.78
Atelectasis	19 (2.2)	2 (1.7)	1.00
Secretion retention	6 (0.7)	0 (0)	1.00
Arrhythmia (A.fib)	58 (6.7)	10 (8.3)	0.52
Arrhythmia (non A.fib)	52 (6)	7 (5.8)	0.93
Myocardial infarction	1 (0.1)	0 (0)	1.00
Cerebral infarction	3 (0.4)	1 (0.8)	0.41
Delirium	39 (4.5)	9 (7.5)	0.16
Seizure	2 (0.2)	0 (0)	1.00
Acute kidney injury	8 (0.9)	1 (0.8)	1.00
Pulmonary thromboembolism	2 (0.2)	1 (0.8)	0.32
Postoperative bleeding	6 (0.7)	1 (0.8)	0.60
Vocal cord paresis	131 (15.2)	20 (16.7)	0.68
Death^a^	1 (0.1)	0 (0)	1.00

Values are presented as *n* (%). ^a^Death within 30 days after surgery. Abbreviations: ARDS acute respiratory distress syndrome, A.fib atrial fibrillation.

**Figure 2:**
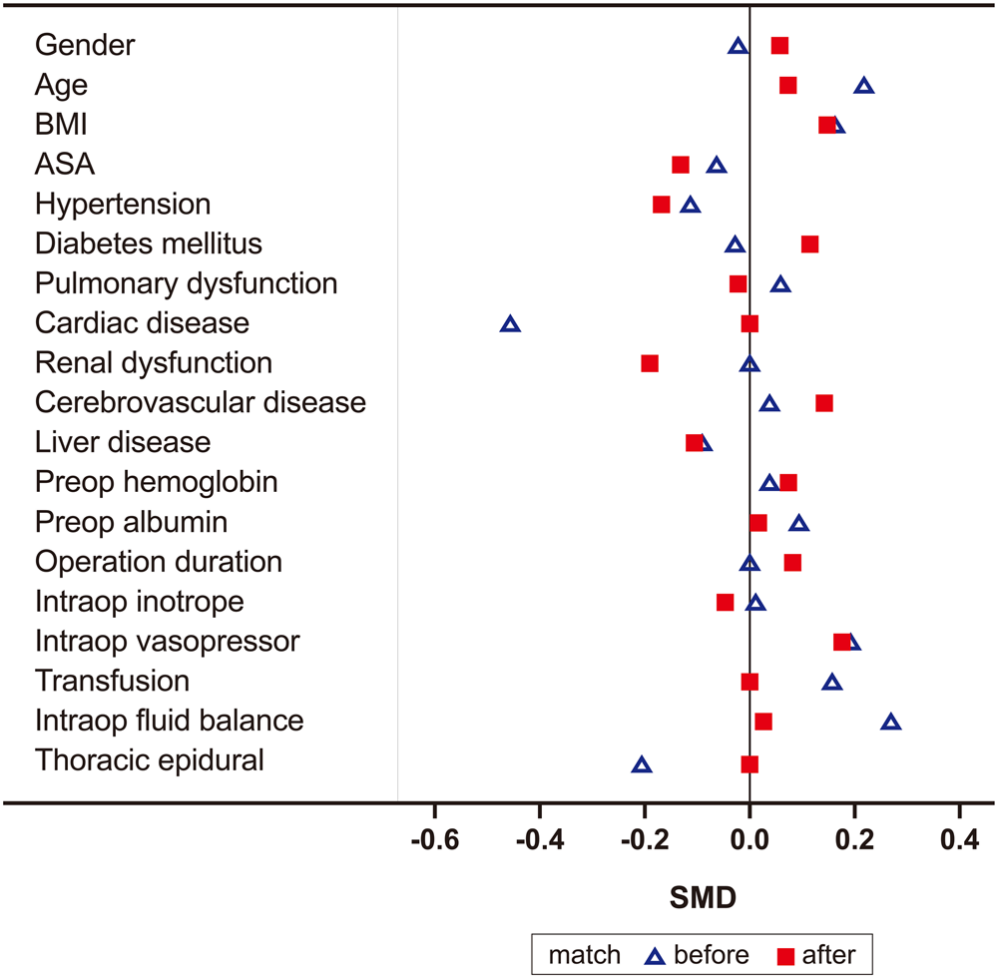
Covariance balance plots of standardized mean differences before (blue triangle) and after (red square) propensity score matching. Matching was successful with all variables’ standardized mean differences <0.2. Abbreviations: BMI body mass index, ASA American Society of Anesthesiologist.

Propensity score matching were performed to adjust the confounders between the two groups. The matching balance is shown in Fig. 2 (*n* = 115 for each group). Propensity score matching revealed that composite complications were not different between the Control and Steroid group (20.0% vs. 21.7%; *P* = 0.75). Among individual categories of complications, ARDS (4.3% vs. 5.2%; *P* = 0.76) and infection (16.5% vs. 15.7%; *P* = 0.86) were not different between the Control and Steroid group, but the incidence of graft dehiscence was higher in the Steroid group than in the Control group (0.9% vs. 7.0%; *P* = 0.0175) (Table 3).

**Table 3:** Postoperative complications between Control and Steroid groups after propensity score matching.

Complication [*n* (%)]	Control (*n* = 115)	Steroid (*n* = 115)	*P* value
Composite complication	23 (20.0)	25 (21.7)	0.75
ARDS	5 (4.3)	6 (5.2)	0.76
Graft dehiscence	1 (0.9)	8 (7.0)	0.0175
Infection	19 (16.5)	18 (15.7)	0.86

Values are presented as *n* (%). Abbreviations: ARDS acute respiratory distress syndrome.

A multivariable logistic regression analysis showed that preventive administration of corticosteroids did not affect the development of composite complications, graft dehiscence, and infection. Instead, age, male sex, lower body mass index (BMI), longer duration of operation, and continuous infusion of vasopressor were independent risk factors (Tables 4, 5 and 6). We could not find risk factors for ARDS (data not shown).

**Table 4:** Multivariable logistic regression for the risk factors of postoperative composite complications.

Variables	Univariable			Multivariable		
	OR	95% CI	*P* value	OR	95% CI	*P* value
Corticosteroids	1.31	0.82–2.09	0.26	1.01	0.58–1.75	0.98
Age > 66 years	1.52	1.09–2.11	0.0125	1.41	0.97–2.06	0.07
Male	1.94	0.95–3.94	0.07	2.67	1.02–6.97	0.0445
BMI ≤21 (kg/m^2^)	1.49	1.06–2.09	0.0215	1.80	1.21–2.67	0.0037
TNM stage 3 & 4	1.51	1.05–2.17	0.0279	1.23	0.83–1.80	0.30
Hemoglobin (g/dl)	0.89	0.82–0.98	0.02	0.98	0.86–1.11	0.74
Albumin (g/dl)	0.58	0.39–0.88	0.01	0.93	0.52–1.67	0.82
Surgery duration, per hour	1.34	1.21–1.49	<0.0001	1.27	1.13–1.43	0.0001
Infusion of inotrope	1.61	1.12–2.31	0.01	1.04	0.68–1.60	0.86
Infusion of vasopressor	1.49	1.06–2.10	0.02	1.35	0.92–1.98	0.12
Transfusion	1.91	1.04–3.50	0.04	0.91	0.42–1.96	0.82

Variables with *P* < 0.2 in univariable analysis were presented in Univariable column. Abbreviations: BMI body mass index, TNM tumor node metastasis, OR odds ratio, CI confidence interval.

**Table 5:** Multivariable logistic regression for the risk factors of postoperative graft dehiscence.

Variables	Univariable			Multivariable		
	OR	95% CI	*P* value	OR	95% CI	*P* value
Corticosteroids	1.43	0.65–3.12	0.37	1.37	0.59–3.20	0.46
BMI ≤ 21 (kg/m^2^)	1.55	0.86–2.78	0.14	1.10	0.56–2.17	0.79
Hemoglobin (g/dl)	0.85	0.73–0.99	0.0375	1.00	0.81–1.23	0.99
Albumin (g/dl)	0.45	0.23–0.90	0.0240	0.69	0.27–1.74	0.43
Hypertension	0.46	0.23–0.91	0.0248	0.55	0.27–1.14	0.11
Surgery duration, per hour	1.71	1.46–1.99	<0.0001	1.57	1.31–1.87	<0.0001
Net balance of fluid	1.00	1.00–1.00	0.0006	1.00	0.46–1.86	0.09
Infusion of inotrope	1.77	0.96–3.25	0.07	0.93	0.46–1.86	0.83
Infusion of vasopressor	2.11	1.11–4.04	0.0234	2.08	1.04–4.17	0.0379
Transfusion	3.00	1.28–7.02	0.0113	1.67	0.62–4.58	0.32

Variables with *P* < 0.2 in univariable analysis were presented in Univariable column. Abbreviations: BMI body mass index, OR odds ratio, CI confidence interval.

**Table 6:** Multivariable logistic regression for the risk factors of postoperative infection.

Variables	Univariable			Multivariable		
	OR	95% CI	*P* value	OR	95% CI	*P* value
Corticosteroids	1.28	0.76–2.18	0.36	1.99	0.53–1.83	0.97
Age > 66 years	2.05	1.41–2.97	0.0002	1.76	1.16–2.68	0.0083
Male	1.76	0.79–3.90	0.16	1.72	0.65–4.55	0.28
BMI ≤ 21 (kg/m^2^)	1.35	0.92–1.99	0.128	1.76	1.13–2.74	0.0130
TNM stage 3 & 4	1.32	0.87–2.00	0.19	1.11	0.71–1.71	0.65
Hemoglobin (g/dl)	0.90	0.81–0.99	0.0330	1.01	0.87–1.18	0.86
Albumin (g/dl)	0.58	0.37–0.92	0.0205	0.95	0.49–1.84	0.87
ASA physical status ≥3	1.84	0.92–3.68	0.08	0.95	0.49–2.71	0.82
Diabetes mellitus	1.52	0.95–2.44	0.08	1.50	0.86–2.62	0.15
Surgery duration, per hour	1.25	1.12–1.40	0.0001	1.18	1.03–1.34	0.0139
Infusion of inotrope	1.61	1.07–2.41	0.0209	1.15	0.71–1.84	0.57
Infusion of vasopressor	1.50	1.02–2.21	0.0406	1.34	0.87–2.07	0.18

Variables with *P* < 0.2 in univariable analysis were presented in Univariable column. Abbreviations: BMI body mass index, TNM tumor node metastasis, ASA American Society of Anesthesiologists, OR odds ratio, CI confidence interval.

## Discussion

In the current study, the preventive administration of corticosteroids did not reduce the incidence of ARDS, nor increase the incidence of infection. However, higher incidence of graft dehiscence was shown in the Steroid group than in the Control group after the confounding factors were adjusted.

Most previous studies confirmed a reduction of pro-inflammatory cytokines with the use of corticosteroids during esophagectomy^15,16,24–26^. However, few studies have proved the clinical effects^5^. The first randomized controlled study (RCT) on this subject was published in 1997 by Takeda *et al*. (*n* = 30)^27^. They reported that no patient developed postoperative complications in the methylprednisolone group compared with five patients in the saline group (*P* = 0.02) in esophagectomy^27^. The largest RCT was published in 2002 by Sato *et al*. (*n* = 66)^15^. They showed that corticosteroids administered 30 min before the surgery reduce interleukin (IL)−6 and IL-8 levels and organ failures (the heart, lung, kidney, and liver, 33% vs. 61%, steroid vs. control)^15^. However, the definition of organ failure was relatively broad in that study^15^. In addition, another RCT published 2 years later (*n* = 40) contradicted their findings, showing no difference in the incidence of post-esophagectomy complications according to the preoperative use of corticosteroids^16^. All of these RCTs included small numbers of patients for the complications analysis and are now outdated.

Several retrospective studies exist on this subject^14,28,29^. They are also mostly small studies (*n* = 36^28^, *n* = 107^29^, and *n* = 234^14^) but reported a reduction of complications known to be related to hyper-inflammation such as systemic inflammatory response syndrome^28^ or ARDS with the preventive use of corticosteroids^14^. The largest retrospective study (*n* = 234)^14^ showed that 125 mg methylprednisolone administered after graft anastomosis reduced C-reactive protein levels and acute respiratory failure (2 vs. 16 patients, steroid vs. control) after esophagectomy^14^.

Only a few meta-analyses have been performed; the latest one, published in 2014, included seven RCTs and four retrospective studies (including three Japanese RCTs not on PubMed). They found no significant differences in the incidence of three categories of postoperative complications between a steroid group and a control group^5^. However, the authors admitted that the studies included were mostly outdated (1994 to 2005), and had low power (17 to 66 patients) and unsatisfactory quality (non-randomized and unblinded studies were included)^5^.

The strength of our study was that we analyzed recent data reflecting current practice, included a large number of patients (*n* = 980) who were treated using a uniform protocol (anesthesia, operation, and perioperative care), and investigated three major complication categories after esophagectomy. Also, the complication categories were defined more comprehensively than in previous studies.

Our results are in line with the 2014 meta-analysis^5^. We did not observe differences between the Steroid and Control group on the incidence of complications. In addition, multivariable analysis showed that the preventive use of corticosteroid was not related to postoperative complications. However, propensity score matching revealed the incidence of graft dehiscence was higher in the Steroid group than in the Control group. Considering inconsistent previous reports and our results, we assume that the administration of corticosteroids is not a major influencing factor on postoperative complications but it may impair would healing compared to no administration under the condition of other risk factors being controlled.

Corticosteroids reduce acute-phase reactants and suppress inflammatory reactions^12,13^. However, the same anti-inflammatory action may be harmful to anastomosis healing^12,17,18^. The secretion of cytokines plays an integral role in successful wound healing^18^. In addition, keratinocyte growth factor expression or responsiveness which is associated with wound-healing, is significantly reduced by glucocorticoid treatment^17^. High levels of pro-inflammatory cytokines reverses inhibitory effect of glucocorticoids on keratinocyte growth factor expression^17^. The National Surgical Quality Improvement Program analyzing 635,265 patients reported wound dehiscence increased 2 to 3-fold with steroid use^12^.

In our study, we administered 100 mg hydrocortisone. Hydrocortisone is a natural glucocorticoid with both anti-inflammatory and mineralocorticoid actions. Most previous studies administered methylprednisolone. Methylprednisolone is a synthetic corticosteroid with no mineralocorticoid effect^30^. No studies have compared the effects of different types of corticosteroids on postoperative complications. We showed that hydrocortisone was also ineffective for preventing ARDS.

Previously, various doses of methylprednisolone (125 mg^14^, 250 mg^28,29^, 500 mg^16^, 10 mg/kg^15^, or 30 mg/kg^27^) were administered. No clear trends are apparent in the efficacy of different doses^5,14,16,26^. For example, 125 mg methylprednisolone was effective for reducing acute respiratory failure^14^. However, in another study, 500 mg methylprednisolone did not affect the frequency of post-esophagectomy complications^16^. The 2014 meta-analysis suggested that high-dose methylprednisolone (up to 30 mg/kg) may be effective for preventing ARDS in its subgroup analysis^5^. However, administering a high dose of corticosteroid may also increase the tendency of graft dehiscence according to our results.

Based on multivariable analysis, we found that preventive administration of corticosteroids did not affect the development of complications. Instead, old age, male sex, lower BMI, longer duration of surgery, and continuous infusion of vasopressor were risk factors for complications. Low BMI and use of vasopressors may be correctable risk factors. Patients with esophageal cancer have the highest incidence of malnutrition (79.8%) among various cancers^31^. Perioperative malnutrition can cause biochemical or immunological abnormalities that are critical to the postoperative healing process and defense against infection^32^. Continuous infusion of vasopressor during esophagectomy was a risk factor for postoperative graft dehiscence. Vasopressors impair blood flow to a graft while providing adequate perfusion to vital organs^33,34^. In a swine model, the esophageal graft experienced severe hypoperfusion after continuous infusion of a vasopressor, especially to subjects in a hypovolemic state^33^. However, use of vasopressor may be a reflection of poor patient condition instead of being a risk factor itself because it is related to other risk factors such as patient’s frailty, bleeding, or low blood pressure from various causes. In addition, longer duration of operation can also be interpreted as more complex operation instead of being a risk factor itself.

This study had some limitations. First, it used a retrospective design, in which inherently uncontrolled factors may have influenced the results. Second, two groups of patients were not recruited in the same time in this study. Although we applied the uniform protocol to treatment during study period, minor changes in the treatment protocol that occurred over time or other time related differences exist. Thus, the potential problems arising from using historical controls may have affected the results. Third, we only focused on the major complications related to corticosteroids, but other complications such as hyperglycemia may also be related to the use of corticosteroids.

In conclusions, the preventive use of corticosteroids did not reduce the incidence of ARDS but may be related to an increased incidence of graft dehiscence. Therefore, routine administration of corticosteroids to prevent ARDS is not recommended due to its lack of apparent benefit.

## Methods

### Ethics approval and consent to participate

This retrospective cohort study was approved by the Institutional Review Board (IRB) of Samsung Medical Center (approval No. 2017-11-004). The IRB waived the need for written informed consent from participants because of non-interventional retrospective design.

### Patient records

Electronic medical records of all patients who underwent esophagectomy from January 2010 to December 2015 in our institute were reviewed (*n* = 1,041). Hydrocortisone 100 mg was routinely administered at the start of operation beginning in April 2015 to prevent ARDS. Patients who received hydrocortisone 100 mg to prevent ARDS during the operation were classified into the Steroid group (patients after April 2015) and patients who did not receive a corticosteroid (patients before April 2015) were classified into the Control group.

Other information collected from the patient’s medical records included age, gender, comorbidities, BMI, American Society of Anesthesiologists (ASA) physical status, duration and type of surgery, preoperative hemoglobin and albumin, intraoperative continuous infusion of an inotrope or vasopressor, perioperative transfusion, net amount of fluid administered during and within 24 hours after surgery, and method of postoperative analgesia. Comorbid conditions included hypertension, diabetes mellitus, renal dysfunction, cerebrovascular disease, cardiac disease, and pulmonary dysfunction. Cerebrovascular disease included a history of cerebral infarction, cerebral hemorrhage, and Parkinson’s disease/dementia/Alzheimer’s disease. Cardiac disease included coronary artery disease and heart failure. Pulmonary dysfunction included lung diseases (i.e., chronic obstructive pulmonary disease, bronchiectasis, asthma, and interstitial lung disease), preoperative forced expiratory volume in 1 second <60% of the predicted value, and current smoker. Current smoker was defined as a patient who was smoking or had stopped smoking within 1 month before the surgery.

### Definition of postoperative complications

Composite complications were the primary endpoint of this study. Three categories of complications up to discharge were included: ARDS, graft dehiscence, and infection. ARDS was defined according to the 2012 Berlin definition as acute (within 1 week of a known clinical insult) hypoxemic respiratory failure (ratio of the partial pressure of arterial oxygen to the fraction of inspired oxygen <300 mm Hg) requiring positive end-expiratory pressure of ≥5 cm H_2_O with bilateral opacities on chest imaging not fully explained by cardiac failure or fluid overload^35^. Graft dehiscence included the development of anastomotic leakage, a significant esophageal fistula, perforation of the bowel or stomach, bronchopulmonary fistula, and graft failure. Infection included pneumonia, empyema, surgical site infection, and catheter-related infection.

### Surgical procedure

The esophageal surgeries included total esophagectomy and total lymphadenectomy with reconstruction using portions of the stomach or colon. Patients underwent esophagectomy via a right thoracotomy, median laparotomy, and/or a bilateral cervical U-shaped incision. The replacement conduit was pulled up through a posterior mediastinal route in all patients. Anastomotic sites were decided based on the tumor level. Three-field lymph node dissection and cervical anastomosis were performed in cases of upper esophageal cancer, and two-field lymph node dissection and intrathoracic anastomosis were performed for mid- and lower-esophageal cancer. The transhiatal approach was performed when a thoracotomy was not required.

### Anesthesia and postoperative management

Anesthesia and postoperative management were performed according to our institutional protocol. Most patients received balanced anesthesia, which was a combination of volatile anesthetic agent, non-depolarizing neuromuscular blocking agent, and a continuous intravenous infusion of remifentanil. The maintenance fluid was lactated Ringer’s solution, infused at a rate of 3–5 ml·kg^−1^·h^−1^. If a volume deficiency was suspected, 5% human albumin (Green Cross Corp., Gyeonggi, Korea) or 6% hydroxyethyl starch (Fresenius Kabi, Seoul, Korea) was infused. A transfusion was performed for effective resuscitation in cases of intraoperative bleeding (transfusion cut-off: hemoglobin <8 g/dl). The protective ventilation protocol was applied to all patients. Mechanical ventilation during one-lung ventilation was maintained with a tidal volume of 5–6 ml/kg predicted body weight at 5 cm H_2_O positive end expiratory pressure. A recruitment maneuver applied to the dependent lung was performed at the commencement of one-lung ventilation and on restarting two-lung ventilation.

All patients stayed in the intensive care unit (ICU) for 2 days. The postoperative analgesic methods were determined according to the surgeon’s preference and contraindications for regional analgesia. Maintenance fluid was administered at a rate of 2–3 ml·kg^−1^·h^−1^. ICU intensivists administered additional fluids based on each patient’s vital signs. Patients were encouraged to ambulate from postoperative day 1 and received a daily physiotherapy program, which included deep-breathing exercises, incentive spirometry, and chest physiotherapy, supervised by physiotherapists and attending nurses during the ICU and ward stays.

### Statistics

Patient demographic and clinical data are summarized as frequencies (percentage) for categorical variables and medians (interquartile range) for continuous variables. The chi-square test was used to compare the incidence of complications between the Control and Steroid groups. The chi-square test or Fisher’s exact test was used to compare other categorical variables between the two groups. The Wilcoxon rank-sum test was used to determine differences in continuous variables between the two groups. Propensity score matching was performed between the two groups to adjust confounding factors. Matched variables were gender, age, BMI, ASA physical status, hypertension, diabetes mellitus, cardiac disease, pulmonary dysfunction, renal dysfunction, cerebrovascular disease, liver disease, preoperative hemoglobin, preoperative albumin, duration of surgery, intraoperative continuous infusion of inotrope or vasopressor, transfusion, fluid balance, and thoracic epidural analgesia. Based on the standard deviation of the logit of the estimated propensity score, one-to-one matching was performed using the nearest-neighbor method with a caliper width of 0.2 in a pairwise manner. The matched data included *n* = 115 for each group. A multivariable logistic regression analysis was used to analyze the risk factors for postoperative complications: univariable analysis was performed for all variables and variables with *P* < 0.2 were further analyzed by multivariable analysis. In all analyses, a two-sided *P* < 0.05 was considered significant. Data were analyzed using SAS 9.4 software (SAS Institute, Cary, NC, USA).

## Data Availability

The datasets generated and analyzed during the current study are available from the corresponding author on reasonable request.
